# Photolysis of 3-Nitro-1,2,4-triazol-5-one: Mechanisms and Products

**DOI:** 10.1021/acsestwater.2c00567

**Published:** 2023-02-02

**Authors:** Hunter W. Schroer, Esteban Londono, Xueshu Li, Hans-Joachim Lehmler, William Arnold, Craig L. Just

**Affiliations:** † Civil & Environmental Engineering, The University of Iowa, Iowa City, Iowa52242, United States; ‡ Occupational & Environmental Health, The University of Iowa, Iowa City, Iowa52246, United States; § Department of Civil, Environmental, and Geo- Engineering, University of Minnesota, 500 Pillsbury Dr. SE, Minneapolis, Minnesota55455, United States

**Keywords:** Nitrotriazolone, insensitive munitions explosives, IMX-101, IMX-104, singlet oxygen, quenchers, bimolecular rate constants

## Abstract

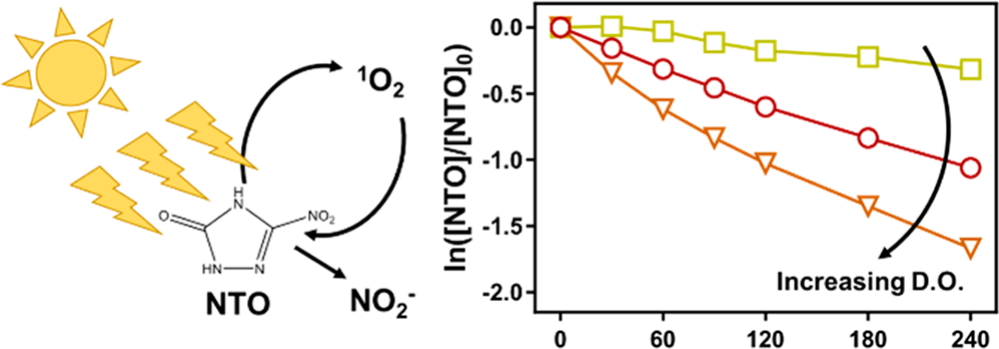

Insensitive munitions formulations that include 3-nitro-1,2,4-triazol-5-one (NTO) are replacing traditional explosive compounds. While these new formulations have superior safety characteristics, the compounds have greater environmental mobility, raising concern over potential contamination and cleanup of training and manufacturing facilities. Here, we examine the mechanisms and products of NTO photolysis in simulated sunlight to further inform NTO degradation in sunlit surface waters. We demonstrate that NTO produces singlet oxygen and that dissolved oxygen increases the NTO photolysis rate in deionized water. The rate of NTO photolysis is independent of concentration and decreases slightly in the presence of Suwannee River Natural Organic Matter. The apparent quantum yield of NTO generally decreases as pH increases, ranging from 2.0 × 10^–5^ at pH 12 to 1.3 × 10^–3^ at pH 2. Bimolecular reaction rate constants for NTO with singlet oxygen and hydroxyl radical were measured to be (1.95 ± 0.15) × 10^6^ and (3.28 ± 0.23) × 10^10^ M^–1^ s^–1^, respectively. Major photolysis reaction products were ammonium, nitrite, and nitrate, with nitrite produced in nearly stoichiometric yield upon the reaction of NTO with singlet oxygen. Environmental half-lives are predicted to span from 1.1 to 5.7 days. Taken together, these data enhance our understanding of NTO photolysis under environmentally relevant conditions.

## Abstract 2

Photolysis of the insensitive explosive NTO forms inorganic nitrogen, singlet oxygen, and hydroxyl radicals, is enhanced by dissolved oxygen, and is predicted to proceed with half-lives of 1−6 days in the environment.

## Introduction

Traditional munitions (e.g., trinitrotoluene (TNT) and hexahydro-1,3,5-trinitro-1,3,5-triazine (RDX)) frequently contaminate soil and water at military testing and training sites with relatively predictable recalcitrance, toxicity, and mutagenicity.^1^ These conventional explosive compounds are now being replaced with new, insensitive formulations to increase soldier safety during storage, transport, and use.^2^ The new compounds, including 3-nitro-1,2,4-triazol-5-one (NTO), have a similar explosive performance to RDX and TNT but are less likely to detonate accidentally. Our full understanding of the safety benefits of NTO is hindered by our limited knowledge of the consequences of environmental releases of NTO and human exposure to NTO. For example, NTO undergoes little to no degradation in aerobic soil and sorbs minimally to soil columns,^3^ leading to potential contamination in groundwater. Under anaerobic conditions, the nitro group of NTO is readily reduced to produce 3-amino-1,2,4-triazol-5-one, both biologically in soil microcosms and by anaerobic wastewater sludges and abiotically by ferrous iron and model humic acids.^3−6^

Moreover, NTO is 166 and 277 times more water-soluble than TNT and RDX, respectively.^3,7−9^ In preliminary studies, the acute toxicity, mutagenicity, and genotoxicity of NTO appear to be lower than those of traditional explosive compounds. Still, NTO caused reproductive effects in male rats at high concentrations.^10−12^ Data also suggest that toxicity from NTO to *Ceriodaphnia dubia* increases 100-fold upon photolysis, despite only a small fraction of NTO being degraded during the examined photolysis period.^13^

If released to surface waters, NTO photolysis is a potential loss process given the minimal biodegradation in soils. Previous results show that, upon photolysis at high concentrations, NTO yields nitrite, nitrate, ammonium, hydroxy-triazolone, and potentially nitric oxide and apparently produces additional unquantified volatile products based on low mass balances after extended irradiation.^14−16^ Based on the high initial concentrations, lack of information on or control of experimental conditions, and unknown reasons for matrix and pH effects, the mechanisms of NTO photolysis have not been fully elucidated.^16−18^ Here, we expand on previous work by evaluating fundamental direct and indirect mechanisms and products of NTO photolysis across a range of pH and initial concentrations to identify relevant parameters and conditions for photolysis of NTO in the environment and identify processes that will influence NTO photolysis in deionized water and environmental matrices.

## Materials and Methods

### Chemicals

Chemicals were obtained commercially and used as received (see Table S1 for source and purity information). NTO was synthesized via 1,2,4-triazolone and characterized as described in section S1.

### Photolysis Experiments

The wavelength-dependent absorbance of a compound and spectral irradiance of the light source, combined with the quantum yield, dictate the direct photolysis rate.^19,20^ In addition, absorbance and quantum yield can change as a function of pH if the compound exhibits acid–base chemistry.^19,20^ Therefore, we collected absorbances of NTO on a spectrophotometer (Hach) in a quartz cuvette with a path length of 1 cm from 100 μM NTO solutions in 5 mM buffer solutions (acetic acid, pH 4–5; phosphate, pH 2–3, 6–8, 12; borate, pH 8.5–11; the buffer identity is not expected to have an effect on light absorption or photolysis rates compared to pH^20^) adjusted to within 0.05 pH units by NaOH or HCl. To determine the acid dissociation constants (p*K*_a_ values) for each protonation state, we conducted a spectrophotometric titration using molar absorptivities at five selected wavelengths (235, 280, 315, 385, and 400 nm). We independently fit each set of pH-dependent wavelength data to eq 1 by assuming the two p*K*_a_ values were independent of one another and minimizing the sum of least-squares for the measured molar absorptivity
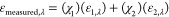
1where χ_i_ is the mole fraction of the *i*th protonation state of NTO, and ε_i,λ_ is the molar absorptivity of the *i*th protonation state of NTO at each wavelength.^20^

Photolysis experiments were conducted at 35 °C using an Atlas Suntest CPS+ solar simulator (Atlas Material Testing Technology) with a xenon lamp and an Atlas UV Suntest filter to simulate the emission spectrum of natural sunlight at an irradiance of 750 W/m^2^. Photolysis solutions contained 5–25 mM buffer (acetic acid, pH 4–5; phosphate, pH 2–3, 6–8, 12; borate, pH 8.5–11). In our preliminary experiments (data not shown), variation in photolysis rates led us to investigate the effect of dissolved oxygen on direct photolysis rate, which indicated that sparging with nitrogen slowed the rate of photolysis, while sparging with oxygen increased the rate of photolysis. Therefore, unless otherwise specified, solutions (10 μM NTO unless noted) were photolyzed in cork-stoppered quartz tubes (outer diameter, 14 mm; inner diameter, 12 mm; volume, 9 mL), held upright at 45° by a test tube rack to prevent contact of the stopper with the solution, after sparging with air for at least 30 s. Aliquots (500 μL) were withdrawn for analysis and stored at 4 °C until analysis. Experiments for direct photolysis rate constants and apparent quantum yield across pH were conducted in duplicate, while remaining experiments were conducted simultaneously with single reactors for each experimental treatment. Foil-wrapped, dark controls indicated that hydrolysis and thermal degradation did not occur from pH 2 to 12 at 35 °C during our experiments (Figure S2). Apparent quantum yield, which is the ratio of transformed molecules to total photons absorbed by the molecules, was determined using an actinometer of *para*-nitroanisole and pyridine using the updated equation found in Laszakovits et al. and detailed in the Supporting Information.^21^

### Probing Mechanisms of Photolysis

To determine direct NTO photolysis mechanisms and to assess self-sensitization due to the production of reactive intermediates, we conducted experiments in solutions of sodium azide (1 mM) or sorbic acid (5 mM) to quench singlet oxygen and triplet excited-state NTO, respectively.^19,22^ These experiments were conducted with 10 μM NTO in 5 mM borate buffer at pH 8.5. Separate solutions were sparged with nitrogen, oxygen, or air for at least 30 s before irradiation. The quartz tubes containing the nitrogen- and oxygen-sparged solutions were sealed with rubber septa to maintain headspace and solution oxygen compositions, and sampling was conducted via syringe by inserting a needle through the septum. In addition, we photolyzed 20 μM furfuryl alcohol (FFA) or 10 μM *para*-chlorobenzoic acid (pCBA), with and without NTO, as a probe for singlet oxygen and hydroxyl radicals, respectively, to quantify the production of these reactive oxygen species (ROS) by NTO.^19,23,24^ Moreover, competitive kinetics experiments between NTO and these two probes were performed with sensitizers to measure the second-order rate constants of NTO with these species (see Supporting Information Section S4). We evaluated singlet oxygen formation from NTO photolysis by irradiating solutions (pH 8.5) containing 20 μM FFA in the presence of varying concentrations of NTO (0, 10, 20, and 30 μM initial NTO), with 1 mM sodium azide (20 μM initial NTO) being used a quencher. We also compared simultaneous NTO photolysis in deuterium oxide (D_2_O) and H_2_O (pH 7.0, pD 7.4, 25 mM phosphate buffer) to evaluate the effect of singlet oxygen on direct photolysis of NTO (10 μM).

**Figure 1:**
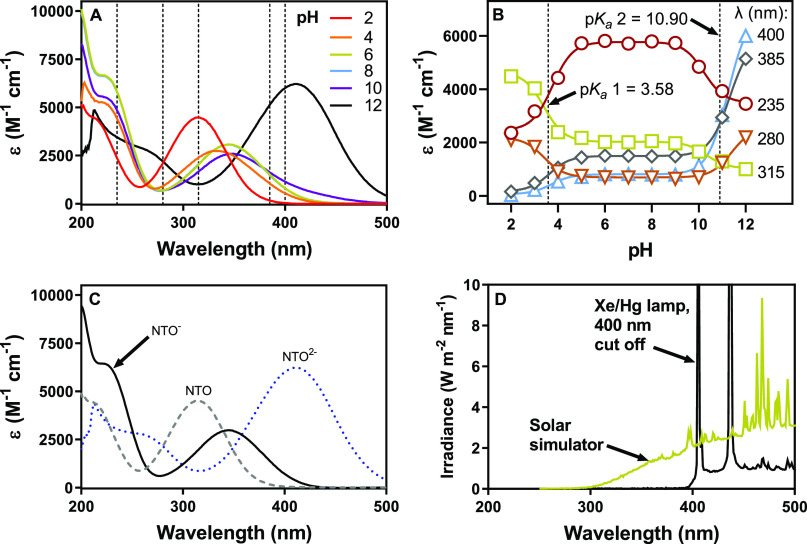
(A) Molar absorptivity of 100 μM NTO from pH 2 to 12. Wavelengths used to determine p*K*_a_ values are indicated with a dashed line. (B) Corresponding molar absorptivities at selected wavelengths and resulting calculated p*K*_a_ values are shown with a dashed line. Solid lines are fit to eq 1. (C) Molar absorptivity of the three protonation states of NTO calculated using matrix deconvolution and (D) spectral irradiance of the solar simulator (750 W/m^2^) and the 200 W Xe/Hg lamp with a 400 nm cutoff filter.

We used a 200 W xenon lamp (Oriel Instruments, Irvine CA, USA) with a 400 nm cutoff filter (Figure 1D) to minimize direct photolysis and added 10 μM Rose Bengal for some experiments to evaluate the effects of singlet oxygen on NTO degradation. To determine transformation products from NTO photolysis under different conditions, we photolyzed 100 μM solutions of NTO in 25 mM buffers at pH 5 sparged with air, pH 9 sparged with air or nitrogen in the solar simulator, and at pH 9, sparged with air, using the lamp with a 400 nm cutoff filter to minimize direct photolysis and maximize singlet oxygen production and subsequent indirect photolysis of NTO.

Solutions of Suwannee River Natural Organic Matter (SRNOM; RO isolate, 2R101N, International Humic Substances Society) were used for indirect photodegradation experiments as a model of aquatic chromophoric dissolved organic matter (DOM).^25,26^ SRNOM solutions were prepared according to Chu et al.,^27^ and frozen aliquots were thawed and used the day of the experiment. The final concentration of the SRNOM stock solution (54.5 mg C L^–1^) was measured using a total organic carbon (TOC) analyzer (TOC-V, Shimadzu) calibrated to a standard solution of potassium hydrogen phthalate (Ricca). We diluted the SRNOM stock solution with deionized water, buffer, and NTO stock solution to create final solutions with environmentally relevant concentrations of SRNOM (0–15.6 mg C/L), 10 μM NTO, and a 5 mM borate buffer, then adjusted the pH to 8.5. Finally, we compared direct photolysis rate constants with initial NTO concentrations ranging from 10^–4^ to 10^–8^ M to assess if the identified mechanisms of NTO photolysis impacted the rate constant of NTO photolysis in 25 mM phosphate buffer adjusted to pH 8.

### Analytical Methods and Data Analysis

We measured NTO, FFA, and pCBA concentrations using high-performance liquid chromatography (HPLC) coupled to an ultraviolet-diode array detector (UV-DAD) or triple quadrupole (QQQ) mass spectrometer (Agilent model 1260 Infinity LCs and UV-DAD, Agilent 6460 Series QQQ, Agilent Technologies). For NTO, we used a Thermo Fisher Acclaim organic acid column and an isocratic eluent of 92% water and 8% acetonitrile containing 0.1% formic acid, measured with detection at a wavelength of 315 nm for UV-DAD or monitoring a mass-to-charge ratio of 129 fragmenting to 55.1 using negative electrospray ionization using the QQQ. See Figure S1 for representative chromatograms. For FFA, we used an Agilent C18 column and an isocratic eluent of 85% water and 15% acetonitrile with UV-DAD detection at 219 nm. For pCBA, the same column, an isocratic eluent of 55% water and 45% acetonitrile, and a wavelength of 234 nm were used. Nitrite and nitrate were measured by ion chromatography (Dionex ICS-2100, Thermo Scientific). Ammonium was measured via derivatization with an *ortho*-phthaldialdehyde (OPA) reagent with excitation at 350 nm and fluorescence at 410–450 nm (Trilogy, Turner Designs). The ammonia sample (0.8 mL) and OPA reagent (0.2 mL) were incubated in a cuvette at room temperature for 2 h before analysis. The OPA reagent consisted of 21 mM borate buffer, 63 μM sodium sulfite, and 50 mL L^–1^ of a 298 mM OPA stock solution in ethanol.

Confidence intervals associated with rate constants were determined from the slope of a linear regression of log-normalized concentration data of pseudo-first-order reactions. Specifically, we multiplied the standard error of the slope by the student’s *t*-value with a 95% confidence level using built-in linear regression and student’s *t*-functions in Microsoft Excel. We determined photolysis rate constants from the slope of a linear regression of log-normalized concentration data of pseudo-first-order reactions using Microsoft Excel and GraphPad Prism 9.4.1. For initial NTO concentration variation experiments, we compared the experimental rate constants using an analysis of variance (ANOVA) test and Tukey’s posthoc test with a 95% confidence level to determine significant differences among treatments (GraphPad Prism). For dissolved SRNOM experiments, we determined if the slope of the simple linear regression was significantly nonzero using an F-test at a 95% confidence level (GraphPad Prism).

## Results and Discussion

### Solution pH and Dissolved Oxygen Content Affect the Rate of NTO Photolysis

We measured the absorbances and calculated the wavelength-dependent molar absorptivity ε of 100 μM NTO in buffered solutions with pH ranging from 2 to 12, as shown in Figures 1A and S3. For the spectrophotometric titration (Figure 1B), we assumed the molar absorptivity of the singly deprotonated state of NTO was the average of the spectra from pH 6, 7, and 8, which left only ε_i_ and p*K*_a_ as the unknown variables to minimize the error function. We calculated the mean and standard error of the mean for each p*K*_a_ to be 3.58 ± 0.08 and 10.90 ± 0.26 (Figure 1B). The p*K*_a_ values are slightly lower than the literature values of 3.76 and 11.25.^28^ Using the calculated p*K*_a_ values, we also determined the NTO component spectra as described previously, which are shown in Figure 1C.^20^ The irradiances of the experimental light sources are shown for reference in Figure 1D.

**Figure 2:**
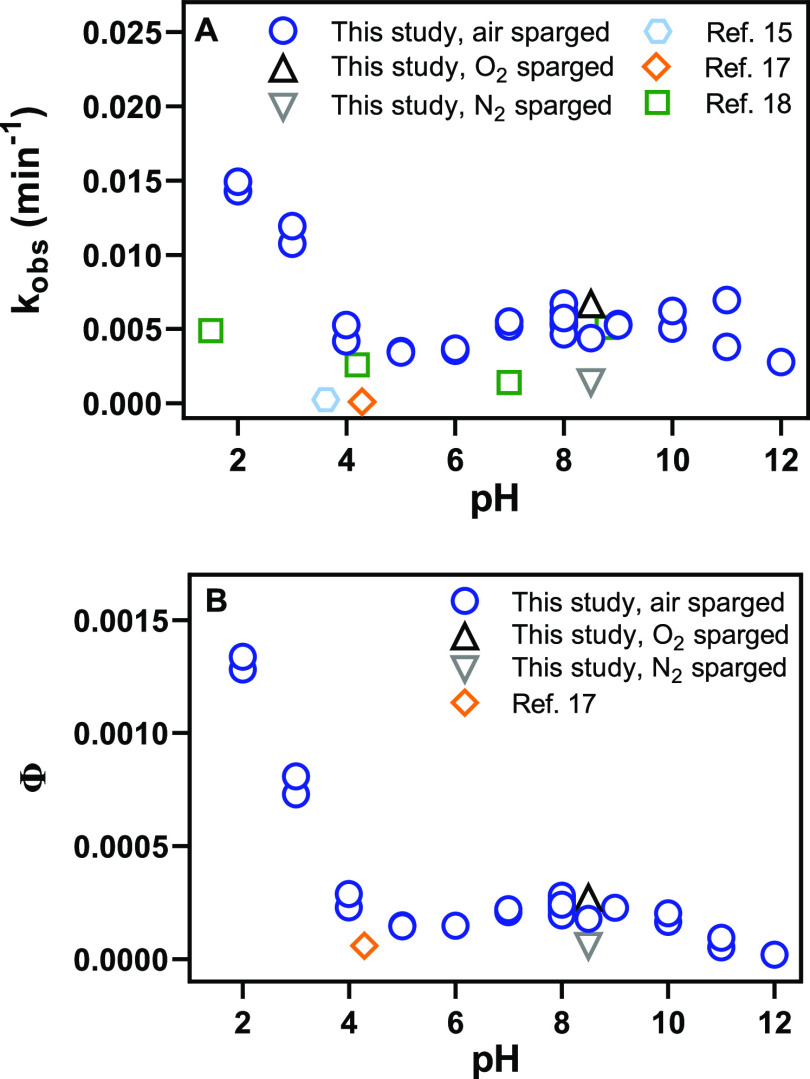
(A) Observed pseudo-first-order degradation rates of direct photolysis of NTO from this study and literature with comparable conditions and (B) apparent quantum yield from 250 to 500 nm (Φ) of NTO from pH 2–12. Each experimental replicate is plotted for reference. At pH 8, the initial concentration of NTO varied from 10^–8^ to 10^–4^ M in this study. Otherwise, the initial concentration of NTO was 10 μM. In this study, 5–25 mM buffers were used, solutions were sparged with air or oxygen/nitrogen as indicated, and illumination was provided by an Atlas Suntest solar simulator set to 750 W m^–2^ total irradiance, 35 °C (Figure 1D). Ref (15) conditions: 223 μM NTO, no buffer, no sparging, SolSim solar simulator (Luzchem Research, Inc., Canada) set to 590 W m^–2^ total irradiance, 25 °C. Ref (17) conditions: ∼10 μM NTO, no buffer, no sparging, outdoors on June 9 to June 13, 2018, latitude of 32°N, ambient temperature. Ref (18) conditions: 7.7 μM NTO, unknown concentration of buffer, no sparging, Atlas Suntest solar simulator 765 W m^–2^, 35 °C. The pH for refs (15) and (17) were not given and were estimated here based on the concentration and calculated p*K*_a_ of 3.58 for NTO in deionized water.

To determine the effect of pH on rates of direct photolysis, we photolyzed NTO in solutions ranging from pH 2 to 12 (Figure 2A). At environmentally relevant pH (6–10), the observed pseudo-first-order rate constants of degradation, *k*_obs_, were similar, in the range of (3.6–5.8) × 10^–3^ min^–1^, resulting in half-lives *t*_1/2_ from 2.1 to 3.2 h. The rate constants we measured in this study were on the same order of magnitude as those of previous studies with similar conditions (Figure 2A).^15,17,18^ However, there is some variability between previously observed rate constants of direct photolysis. Others observed slower rate constants of direct photolysis in their experiments than those observed here, likely due to a lower total irradiance.^15,17^ Indeed, our predicted environmental rate constants of direct photolysis agree well with the published rate constants, as described below in the Environmental Significance section. In addition, the reactors in the two previous studies did not contain buffers.^15,17^ The initial pH of the solutions was not measured in the earlier studies and was estimated here based on the acidity of NTO. Our results generally agreed with additional previous work utilizing the same solar simulator.^18^ Still, this prior work used a higher total irradiance setting for their experiments and measured lower rate constants of direct photolysis. The range in direct photolysis rate constants observed when sparging with nitrogen versus oxygen (see Figure 2A, pH 8.5) could explain some of the variations among observations in previous photolysis studies.

We calculated the apparent quantum yield for NTO from pH 2 to 12 for air-saturated solutions (Figure 2B). Using the results, we calculated the component quantum yields (Table 1) using the component spectra.^20,21^ Apparent quantum yields ranged from 2.0 × 10^–5^ at pH 12 to 1.3 × 10^–3^ at pH 2 and ranged from 1.5 × 10^–4^ (pH 6) to 2.4 × 10^–4^ (pH 8) in the environmentally relevant range. Below pH 5, the rate of photolysis and the apparent quantum yield were dramatically higher, attributed to the protonation of NTO below its first p*K*_a_, predominantly yielding the neutral species at lower pH (Table 1). The increase in the observed reaction rate and apparent quantum yield at low pH could also be due to an increase in hydroxyl radical formation from nitrite at low pH (p*K*_a_ ≈ 3.3).^29^ We did not calculate the potential contribution of the nitrite–nitrous acid system to the pseudo-first-order photodegradation of NTO because the associated low pH values are not environmentally relevant. Sparging with nitrogen decreased the apparent quantum yield of NTO, while sparging with oxygen increased the apparent quantum yield of NTO (Figure 2B, pH 8.5). We did not measure or quench any potential residual oxygen during the nitrogen sparging experiments, so this condition represents a qualitative decrease rather than the complete elimination of dissolved oxygen. The quantum yield reported in a previous study agrees more closely with our results than the direct photolysis rate, which is likely lower than our results because our reactor was saturated with air (Figure 2).^17^

**Table 1:**
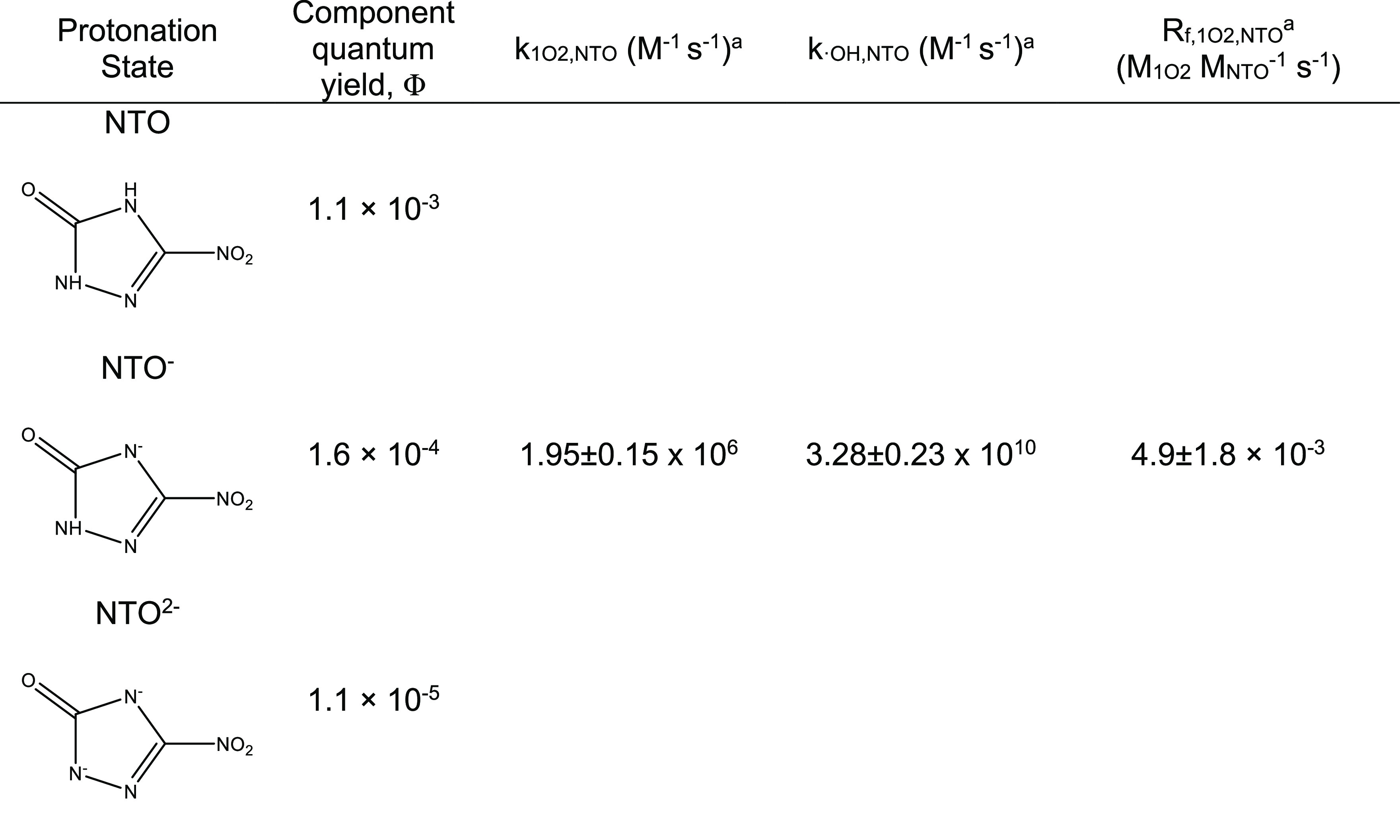
Apparent Quantum Yield for Each Component of NTO Assuming Air-Saturated Conditions, Bimolecular Rate Constants for Hydroxyl Radicals and Singlet Oxygen with NTO, and Formation Rate of Singlet Oxygen from NTO

a Errors for rate constants are 95% confidence intervals for the slope of a linear regression of experimental data.

### Mechanisms of Direct and Indirect Photolysis

**Figure 3:**
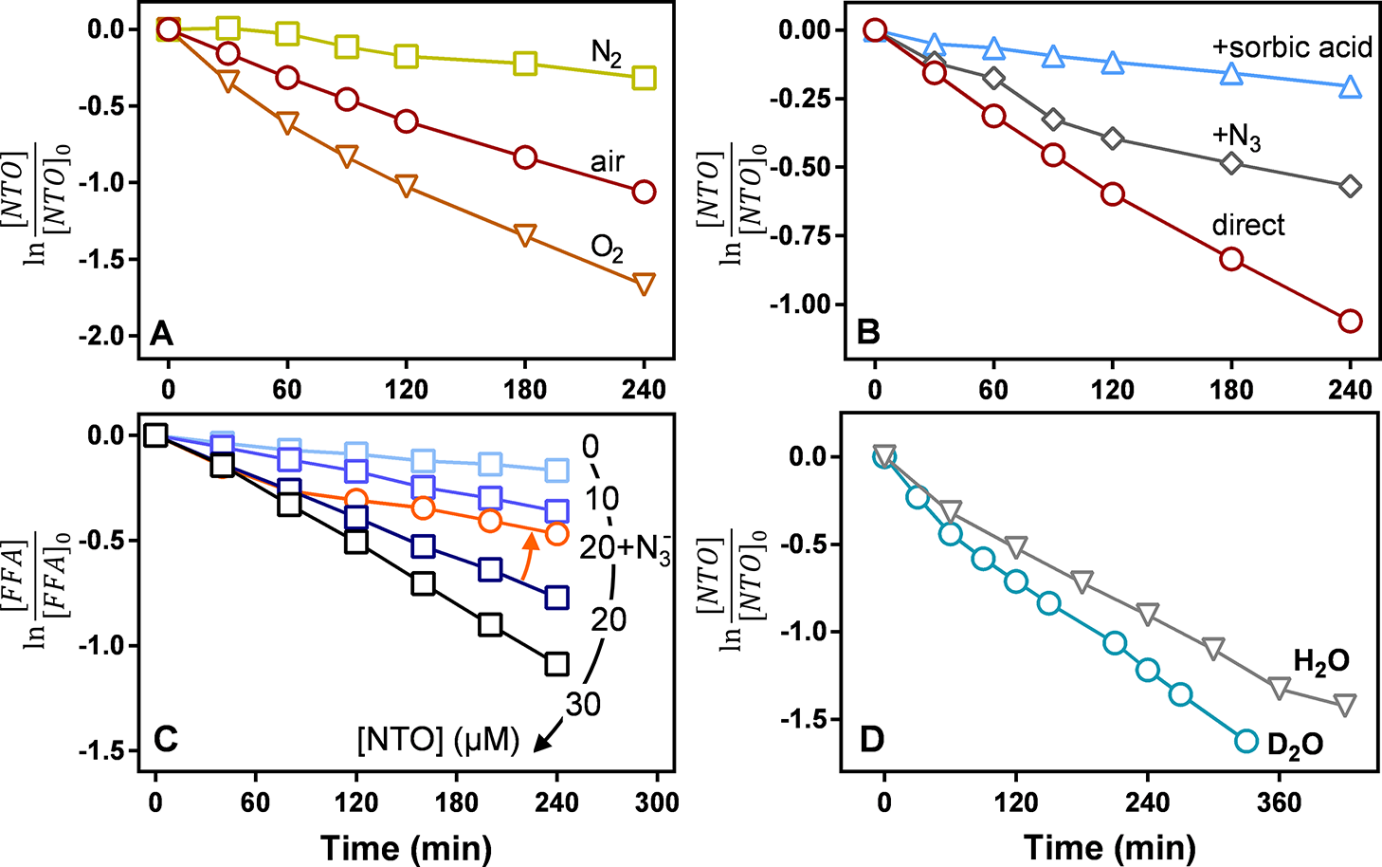
Probing mechanisms of NTO direct photolysis. (A) Direct photolysis of NTO (10 μM) with varied dissolved oxygen regimes. (B) Direct photolysis of NTO (10 μM) with and without quenchers of singlet oxygen (1 mM sodium azide, N_3_^–^) or excited triplet states (5 mM sorbic acid). Production of ^1^O_2_ by NTO as demonstrated by (C) photodegradation of 20 μM FFA in the presence of varying concentrations of NTO and 1 mM sodium azide (pH 8.5) and (D) photolysis of NTO in D_2_O and H_2_O (pH 7, pD 7.4).

We used several methods to probe the factors affecting the rate of direct photolysis, including variable oxygen saturation and quenchers of specific reactive species. As mentioned, an increase in dissolved oxygen increased the rate of NTO photolysis (Figure 2A, Figure 3A). We found that azide and sorbic acid slowed the direct photolysis of NTO (Figure 3B). The dramatic decrease in NTO direct photolysis with sorbic acid present suggests that NTO photolysis proceeded through a triplet excited state,^22^ which agrees with the pathway proposed by a previous computational study.^30^ Direct photolysis of NTO was slower with azide present, suggesting that singlet oxygen is involved because azide is known to physically quench singlet oxygen.^19,26^ Because singlet oxygen is formed from triplet excited states, sorbic acid quenches both reactions mediated by triplet excited states and prevents singlet oxygen formation.^31^

Both N_2_ sparging and azide quenching to eliminate singlet oxygen formation have confounding effects.^31^ First, the sodium azide added (1 mM) is only estimated to quench 60% of singlet oxygen formed, which means that singlet oxygen is only reduced, not eliminated, under these conditions.^25^ In the case of N_2_ sparging, less dissolved O_2_ suppresses singlet oxygen formation. However, because O_2_ also quenches triplet excited-state dissolved organic matter (^3^DOM*), less dissolved O_2_ potentially eliminates a major sink for triplet excited-state NTO (^3^NTO*) relaxations to NTO and potentially facilitates faster degradation of ^3^NTO* to alternative products. Because the rate of direct photolysis was slower with N_2_ sparging, oxygen presumably plays a more important role as a source of ROS than a sink of ^3^NTO* during direct photolysis. Similar results have been observed for photolysis of the phytoestrogens genistein and daidzein, which were quenched by sorbic acid in deionized water, and the reaction also slowed under deoxygenated conditions.^19^

Once we hypothesized that NTO sensitizes ROS formation upon irradiation, we further evaluated the role of singlet oxygen and hydroxyl radical in the direct photolysis of NTO. Photolysis of NTO has been shown to produce nitrite and nitrate, which can each subsequently generate hydroxyl radicals upon irradiation.^15,16,19^ Therefore, we quantified the bimolecular rate constant of NTO reacting with hydroxyl radicals using a competition kinetics approach (Table 1, Figure S5, Section S4). The calculated bimolecular rate constant of the reaction of NTO with hydroxyl radicals was (3.28 ± 0.23) × 10^10^ M^–1^ s^–1^, which is at the diffusion-controlled limit.^32^

To investigate singlet oxygen formation from NTO photolysis, we irradiated FFA with varying initial amounts of NTO and sodium azide in an aqueous solution (Figure 3C). The presence of NTO dramatically enhanced the degradation of FFA, a well-characterized probe for singlet oxygen degradation.^19,24,25^ Based on the reaction rate of FFA with NTO, we calculated a production rate of singlet oxygen from NTO according to the following equations
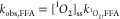
2
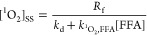
3where [^1^O_2_]_ss_ is steady-state concentration of singlet oxygen, *k*_^1^*O*_2,FFA__ = 1.273 × 10^8^ M^–1^ s^–1^ at 35 °C, *k*_obs,FFA_ is the experimentally observed degradation rate (s^–1^), *R*_f_ is the singlet oxygen formation rate (M s^–1^), *k*_d_ is the singlet oxygen deactivation rate constant in water (2.76 × 10^5^ s^–1^), and [FFA] is initial concentration of FFA.^23^ Simplifying eq 3 because *k*_d_ ≫ *k*_^1^*O*_2,FFA[FFA]__ and substituting for [^1^O_2_]_ss_ yields *R*_f_ as follows.
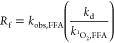
4We plotted *R*_f_ against the initial concentration of NTO in the experiments and calculated the slope, yielding a singlet oxygen production rate of (4.9 ± 1.8) × 10^–3^ M_1O2_ M_NTO_^–1^ s^–1^ (Figure S4, Table 1). FFA is also known to react with hydroxyl radicals, so we conducted a similar experiment with pCBA, a probe for hydroxyl radicals. Overall, the concentration of pCBA was mostly unchanged in the presence or absence of NTO, suggesting that FFA degradation was predominantly due to singlet oxygen derived from NTO rather than hydroxyl radicals (see section S2 for further details). This finding was complemented by an observed decrease in FFA degradation with the addition of azide, which quenches singlet oxygen (Figure 3C). Once again, at a concentration of 1 mM azide, singlet oxygen is expected to be reduced, rather than eliminated.^25^

In addition to producing singlet oxygen, NTO is degraded by singlet oxygen. We conducted an experiment to determine the second-order, bimolecular rate constant of NTO with singlet oxygen using Rose Bengal as a sensitizer and FFA as a probe and found an estimated rate constant of (1.95 ± 0.15) × 10^6^ M^–1^ s^–1^ (Table 1, Figure S5, Section S4). Because NTO itself reacts with singlet oxygen, it was not surprising that sparging the solution with O_2_ prior to irradiation increased the direct photolysis rate of NTO and that removing oxygen with N_2_ sparging slowed the reaction. To further confirm singlet oxygen formation and degradation of NTO, we also photolyzed NTO in deuterium oxide (D_2_O) (Figure 3D). Water quenches singlet oxygen, and D_2_O is often used to extend the lifetime of singlet oxygen molecules.^24^ The reaction in D_2_O was faster than in H_2_O, further confirming that direct photolysis of NTO produces singlet oxygen (*p* < 0.001, F-test comparing pseudo-first-order rate constants).

To our knowledge, NTO has not been released to surface waters, but this would likely occur at dilute concentrations, similar to RDX being detected in surface waters near demolition sites (up to 3 μg/L or 0.02 μM).^33^ Compared to naturally present reactive species and scavengers in environmental waters, production of singlet oxygen (from NTO self-sensitization) and hydroxyl radicals (from NTO-derived nitrite and nitrate) is expected to be negligible at environmentally relevant NTO concentrations. For example, in our experimental setup (10 μM NTO), singlet oxygen is predicted to have an initial (maximum) steady-state concentration of 1.8 × 10^–13^ M and contribute a pseudo-first-order reaction rate of 2.1 × 10^–5^ min^–1^ (∼0.5% of the observed rate) using eqs 2 and 3 adapted to NTO rates and concentrations. Similarly, if we make broad assumptions that 10 μM NTO yields 5 μM of nitrite and nitrate, the reaction between NTO and nitrite (*k*_OH,NO2_ = 1.1 × 10^10^ M^–1^ s^–1^)^34^ is the only hydroxyl radical loss process, and a quantum yield of hydroxyl radicals of 0.03 from nitrite and 0.01 from nitrate photolysis, respectively^34^ (using published molar absorptivities for nitrite and nitrate),^35^ we calculate a pseudo-first-order reaction rate of 3.6 × 10^–4^ min^–1^ (∼8% of the direct rate) between NTO and hydroxyl radical. Note that the assumptions listed above would result in a singlet oxygen reaction rate directly proportional to the concentration of NTO, while the hydroxyl radical reaction rate is independent of initial NTO (nitrite concentration is half that of NTO). If higher concentrations of nitrite are naturally present in surface waters, hydroxyl radical formation is expected to contribute to faster NTO degradation (e.g., 23% of the direct rate when nitrite concentration is double that of NTO). At higher NTO concentrations in a previous laboratory study, produced singlet oxygen and hydroxyl radical likely impacted observed photolysis rates.^16^

### Effect of pH, Indirect Mechanisms, and Sparging with Nitrogen on NTO Photoproducts

**Figure 4:**
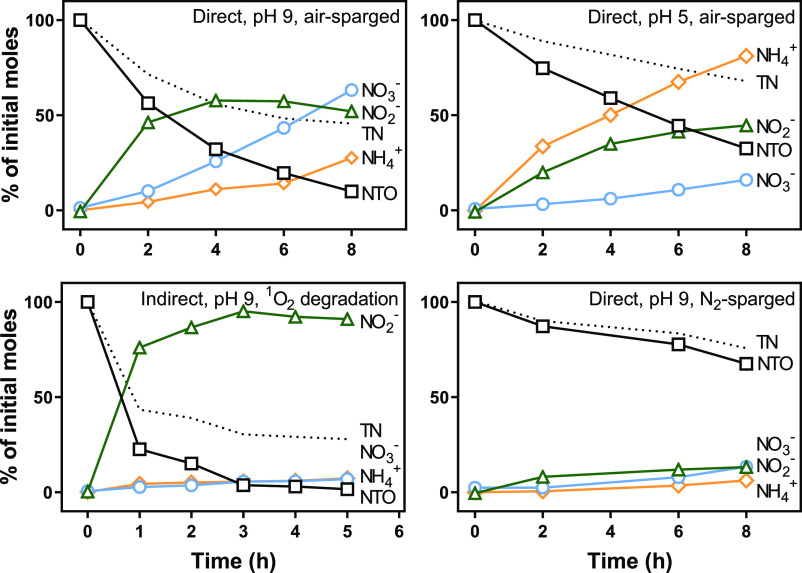
Photolysis and inorganic nitrogen products from photolysis of ∼100 μM NTO under varying conditions (25 mM buffers). Total nitrogen balance is labeled “TN.”

In terms of transformation products, we observed that NTO photolysis produced nitrite, ammonia, nitrate, and trace levels of hydroxy-triazolone, consistent with other studies (Figure 4).^14−16^ Because hydroxy-triazolone did not accumulate during the photolysis, we photolyzed hydroxy-triazolone directly and with initially added nitrite to determine if nitrite facilitated indirect photolysis of hydroxy-triazolone (Figure S6). Not only was hydroxy-triazolone directly photolyzed, but nitrite greatly increased the rate of hydroxy-triazolone loss, providing a rationale for the observation that hydroxy-triazolone was only present in low concentrations as a reactive intermediate. In addition to direct and nitrite-mediated indirect photolysis, hydroxy-triazolone was degraded quickly by singlet oxygen, further explaining the observed lack of accumulation (Figure S7). Adding excess initial nitrite did not affect NTO photolysis and even slowed the reaction when 5 mM was added (Figure S6), which was likely due to light screening by nitrite.

To further delineate the mechanisms of NTO photolysis, we compared the inorganic nitrogen products formed under varying conditions, including pH, dissolved oxygen, and singlet oxygen degradation (Figure 4). The results indicated that nitrite is a prominent product of NTO photolysis. When subjected to singlet oxygen degradation, NTO produced nitrite in an almost stoichiometric yield, with little nitrate and ammonium formation. Under direct photolysis conditions, more nitrate and ammonium were formed than by singlet oxygen alone. Nitrite can be photo-oxidized to produce nitrate.^36^ Apparently, the primary direct photolysis products of NTO are also subject to direct photolysis to form ammonium, consistent with density functional theory predictions.^30^ The higher concentration of ammonia observed at pH 5 compared to pH 9 could have been due to the volatilization of ammonia at pH 9. When the solution was initially sparged with nitrogen gas, the rate of photolysis was much slower, and less inorganic nitrogen was formed, even when accounting for the amount of NTO degraded. This finding indicates that the direct, oxygen-free photolysis reaction could form other unknown products.

### Effect of Initial Concentration and Dissolved Organic Matter on Rate of NTO Photolysis

**Figure 5:**
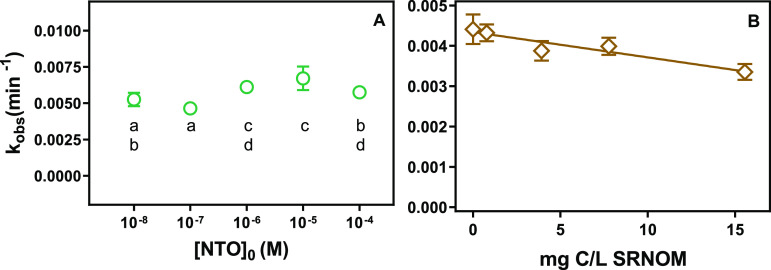
(A) Observed pseudo-first-order rate constants of NTO direct photolysis with initial concentrations spanning 5 orders of magnitude (pH 8, 25 mM phosphate buffer). A one-way ANOVA determined a statistical significance between rates (*p* < 0.001), and lowercase letters indicate significantly different groups by a posthoc Tukey multiple comparisons test (α = 0.05). (B) Rates of indirect photolysis of NTO with respect to SRNOM concentration (10 μM NTO, 5 mM borate buffer, pH 8.5). Rates are corrected for light screening; error bars are 95% confidence intervals and are not displayed when within the symbols. The solid line in (B) is a linear fit to the data (slope = −6.3 × 10^–5^ (L min^–1^ mg C^1–^), intercept = 4.3 × 10^–3^ min^–1^, *r*^2^ = 0.90).

To determine the effect of more environmentally relevant initial concentrations of NTO on the observed rate of photolysis, we exposed NTO solutions ranging from 10 nM to 100 μM (1.3–13 000 μg/L) to simulated sunlight. Even at the lower concentrations, the observed rate was similar among all concentrations tested (Figure 5A). An ANOVA analysis revealed significant differences among rates (*p* < 0.001), but the rate at the lowest concentration (10 nM) was not significantly different from the rate at the highest concentration (100 μM) according to a posthoc Tukey multiple comparisons test. The lack of trend as a function of concentration suggests that the rate of NTO photolysis in surface waters will not be substantially affected by initial NTO concentration. The similarity in reaction rates at low concentrations may indicate that the rate-limiting step of photolysis is the absorbance of a photon or intersystem crossing to a triplet state before further reactions. Therefore, different mechanisms (e.g., direct photolysis vs singlet oxygen degradation) may occur at lower concentrations than higher ones. At the same time, the overall rate remains similar due to a rate-limiting, concentration-independent step. Further work would be needed to confirm this hypothesis (e.g., investigating photoproduct distributions at various concentrations).

To evaluate the impact of dissolved organic matter on NTO photolysis, we photolyzed NTO in solutions of SRNOM that represent a range of DOM concentrations found in natural waters (0–15.6 mg C/L). As the concentration of SRNOM increased, the rate of NTO photolysis decreased, even when the rate was corrected for light screening (Figure 5B). The slope of the linear regression was significantly nonzero when evaluated at a 95% confidence level using an F-test (*p* = 0.042). The observed decrease in NTO degradation indicates that indirect photolysis mediated by DOM does not enhance observed rates of NTO photolysis. This result could be due to the antioxidant properties of SRNOM (i.e., the SRNOM may reduce intermediates of NTO photo-oxidation).^37−40^ For example, tryptophan photolysis proceeds through a radical cation that is quenched by NOM.^41^ A similar process could happen with NTO, where redox-active moieties quench a radical intermediate or the produced singlet oxygen in the SRNOM. In addition, close physical proximity to DOM, as evidenced by hydrophobicity of probe molecules, has been shown to dramatically increase reactivity of singlet oxygen^42^ and hydroxyl radicals^43^ produced by irradiation of DOM. Due to the hydrophilic nature of NTO, we expect sorption of NTO to DOM to be minimal, which further explains the lack of NTO degradation in SRNOM solutions.

### Environmental Significance

**Figure 6:**
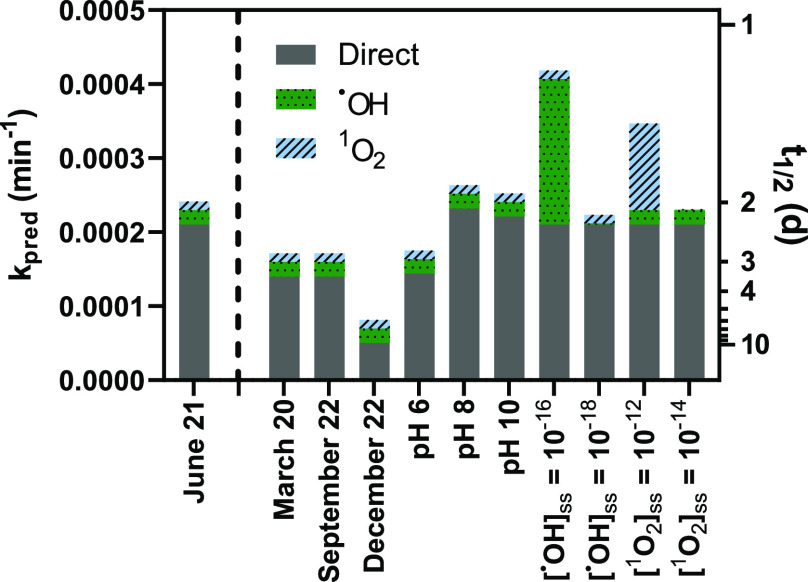
Predicted environmental photolysis rates *k*_pred_ and half-lives *t*_1/2_ of NTO under varying environmental conditions. The “base case” is shown on the left (June 21 daily average irradiance, latitude 40° N; pH 7; steady-state hydroxyl radical concentration [·OH]_ss_ is set to 10^–17^ M; steady-state singlet oxygen concentration [^1^O_2_]_ss_ is set to 10^–13^ M). As specified on the *x*-axis, individual values in the base case were altered, and predicted photolysis rates and half-lives are shown for each season as well as along a range of pH, [·OH]_ss_, and [^1^O_2_]_ss_ values that are common in natural waters.^24,44−46^

Using the apparent quantum yield, molar absorptivities, and bimolecular reaction rates for NTO, we calculated predicted environmental photolysis rates *k*_pred_ according to eq 6
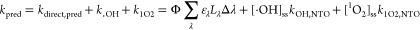
6where Φ is the calculated apparent quantum yield, ε_λ_ is the molar absorptivity at a specific wavelength, *L*_λ_ is the day-averaged irradiance at a specific wavelength at 40° N from Apell and McNeill,^47^ Δλ is equal to one, [·OH]_ss_ is the steady-state concentration of hydroxyl radical, and *k*_i,NTO_ values are listed in Table 1. The predicted summer-time direct photolysis degradation rate at 40° N latitude was found to be 2.1 × 10^–4^ min^–1^, which translates to a half-life of 2.3 days (Figure 6). The seasonal variation of predicted photolysis of NTO is as expected: lower and similar rates are predicted in the spring and fall compared to summer. At the same time, the winter should yield the lowest rate of NTO photolysis. Under average hydroxyl radical (10^–17^ M) and singlet oxygen (10^–13^ M) concentrations,^44−46^ direct photolysis dominates the predicted degradation of NTO. However, in waters with concentrations of hydroxyl radicals (e.g., containing high nitrate) or singlet oxygen near the higher end of those observed in natural waters, the indirect mechanisms could contribute substantially to NTO degradation. For example, water containing a steady-state concentration of 10^–16^ M hydroxyl radical is predicted to contribute 47% of the predicted overall NTO photolysis rate, resulting in a total degradation rate of 4.2 × 10^–4^ min^–1^ and a half-life of only 1.1 day (Figure 6). Based on our results with model natural organic matter, the predicted rates are likely higher than what may be observed, as SRNOM slowed the rate of NTO photolysis (Figure 5B).

## Conclusions

Given the predicted results, NTO photolysis (including direct and indirect pathways) is expected to yield a minimum half-life of 1.1–5.7 days over a range of typical surface water conditions, assuming shallow water and air-saturated conditions. The rate of NTO photolysis would vary with pH, season, and latitude, and the photodegradation rate is mostly governed by direct photolysis under typical conditions. Taken together, these data enhance our understanding of NTO photolysis mechanisms, products, and predicted half-lives, which would likely dominate NTO degradation in aerated surface water given the minimal aerobic biodegradation and sorption of NTO.

## Supporting Information Available

Additional experimental details, light screening and quantum yield calculations, second-order rate constants for singlet oxygen and hydroxyl radical, contribution of NTO-derived hydroxyl-radical mediated degradation of FFA (PDF)
