# Supplementary material for: Photolysis of 3-Nitro-1,2,4-triazol-5-one: Mechanisms and Products
Source: ACS ES T Water. 2023 Feb 2;3(3):783–92. doi: 10.1021/acsestwater.2c00567 (PMC10012174; doi:10.1021/acsestwater.2c00567)
Supplement: Supplementary file 1 — ew2c00567_si_001.pdf [file ew2c00567_si_001.pdf]

SUPPORTING INFORMATION

Photolysis of 3-nitro-1,2,4-triazol-5-one: mechanisms and products

Hunter W. Schroer†, Esteban Londono†, Xueshu Li‡, Hans-Joachim Lehmler‡, William Arnold§, and Craig L. Just†*

†Civil & Environmental Engineering, The University of Iowa, Iowa City, Iowa 52242, United States
‡Occupational & Environmental Health, The University of Iowa, Iowa City, Iowa 52246, United States
§Department of Civil, Environmental, and Geo- Engineering, University of Minnesota, 500 Pillsbury Dr. SE, Minneapolis, MN, 55455, USA;
*Email: hunter-schroer@uiowa.edu. Phone: 319-335-5051. Fax: 319-335-5660.

Table of Contents

Section S1. Chemicals and Synthesis and Characterization of 3-Nitro-1,2,4-Triazol-5-one (NTO) 3

Section S2. Contribution of NTO-derived, hydroxyl-radical mediated degradation of FFA 5

Section S3. Light screening and quantum yield calculations 6

Section S4. Singlet oxygen and hydroxyl radical second-order rate constants 7

References 19

Table of Figures

Table S1. Source, purity, and abbreviations of chemicals utilized. 3

Scheme 1. Preparation of 3-nitro-1,2,4-triazol-5-one (**3**, NTO) via one-pot synthesis of TO 3

Figure S1. Representative chromatograms depicting NTO detection (using HPLC with UV-DAD monitoring at 315 nm) and degradation through five hours of photolysis (pH 2, 10 μM NTO initial concentration). 9
Figure S2. Experimental data comparing illuminated and foil-wrapped dark controls. Data are the natural log of the concentration of NTO at each time point normalized to the initial concentration of NTO. 10
Figure S3. A) molar extinction coefficient of NTO from A) pH 2 to 6 and B) pH 7 to 12 (100 μM NTO), 25 mM buffer solution 10
Figure S4. Calculated formation rate of singlet oxygen calculated from FFA degradation rates as a function of initial concentration of NTO. 11
Figure S5. Determination of 2nd-order reaction rate constants of NTO with A) singlet oxygen using FFA as a probe and B) hydroxyl radical using competition kinetics with pCBA as a probe and nitrate as a sensitizer.[7] The estimated 2nd-order, bimolecular reaction rate constant of NTO with singlet oxygen was 2.0×10^6 M^{-1} $s^{-1}$and with hydroxyl radicals was 3.3×10^{10} M^{-1} s^{-1}. Solid lines are linear regression and dotted lines are 95% confidence intervals. 12
Figure S6. Effect of nitrite on 10 μM A) NTO and B) hydroxyl-triazolone photolysis (25 mM, pH 9 borate buffer, n=1). 13
Figure S7. Indirect photolysis of hydroxyl-triazolone (25 μM, 25 mM pH 9 borate buffer, n=1) with 10 μM Rose Bengal to generate singlet oxygen under a 200W Xe-Hg lamp with a 400 nm cut-on filter. Asterisks indicate non-detect (LOD < 0.1 μM). 13
Figure S8. ^{1}H NMR of 1,2,4-triazolone (TO) 14
Figure S9. ^{13}C NMR of 1,2,4-triazolone (TO) 15
Figure S10. ^{1}H NMR of 3-nitro-1,2,4-triazol-5-one (NTO) 16
Figure S11. ^{13}C NMR of 3-nitro-1,2,4-triazol-5-one (NTO) 17
Figure S12. Example high resolution mass spectra and elemental composition report of synthesized 3-nitro-1,2,4-triazol-5-one (NTO). Report was an average of three mass spectra (*m/z* 129.0048, 129.0050, and 129.0050). 18

Section S1. Chemicals and Synthesis and Characterization of 3-Nitro-1,2,4-Triazol-5-one (NTO)

Table S1. Source, purity, and abbreviations of chemicals utilized.

Compound	Also known as	Purity	Source
Rose Bengal	RB	certified Biological Stain	Fisher Scientific
furfuryl alcohol	FFA	98%	Sigma Aldrich
para-chlorobenzoic acid	pCBA	99%	Acros Organics
o-phthalaldehyde	OPA	99.1%	MP Biomedicals
2,4-hexadienoic acid	sorbic acid	certified reference material	Sigma Aldrich
urazole (3-hydroxyl-1,2,4-triazol-5-one) CAS 3232-84-6	hydroxyl-triazolone	>98%	TCI America
sodium azide	N_3	>99.5%	Sigma Aldrich
deuterium oxide	D_2O	100% isotope	Cambridge Isotope Labs

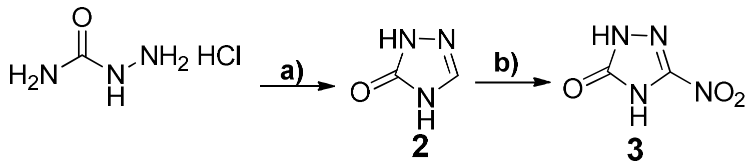

Scheme 1. Preparation of 3-nitro-1,2,4-triazol-5-one (**3**, NTO) via one-pot synthesis of TO. a) HCO_2H, reflux 2h, recrystallized from ethanol, yield 93%; b) Fuming HNO_3, 70 ºC, overnight, yield 42%.

After synthesis of 1,2,4-triazolone (TO) and 3-nitro-1,2,4-triazol-5-one (NTO) as shown in Scheme 1 (details below), NMR spectra (^{1}H and ^{13}C) were recorded on a Bruker Avance DRX-400 spectrometer in the University of Iowa Central NMR Research Facility (Iowa City, IA,

USA). DMSO-d_6 was used as the solvent for NMR. High resolution mass spectra were collected at the High Resolution Mass Spectrometry Facility at the University of Iowa on a Waters Q-TOF Premier mass spectrometer via direct infusion of an NTO solution in water using negative electron spray ionization. A solution of leucine enkephalin was infused as a lock mass, a mass to charge (m/z) range of 90−1000 was monitored with a scan rate of 0.5 s/scan and a sampling cone voltage of 15. The desolvation gas (350 °C) flow was 650 L/h and the capillary voltage was 2.5 kV. Three consecutive spectra were collected and averaged, and the built-in Waters Elemental Composition Report (single mass analysis) software was used to predict the elemental composition with a mass tolerance of 5.0 mDa (Figure S10).

Synthesis of 1,2,4-triazolone (TO)

Semicarbazide hydrochloride (16.7 g, 0.15 mol) was added to a stirred solution of formic acid (24 mL). After the semicarbazide hydrochloride had dissolved completely, the reaction mixture was heated under reflux for 2 hours. Excess formic acid was removed by filtration under reduced pressure and the crude product was recrystallized from ethanol to obtain 12 g (crude yield: 94%) TO as a white solid. ^1H NMR (400 MHz, DMSO-d_6) δ 11.32-11.42 (br s, 2H), 7.70 (s, 1H); ^{13}C NMR (100 MHz, DMSO-d_6) δ 165.4, 145.7.

Synthesis of 3-nitro-1,2,4-triazol-5-one (NTO)

Due to safety concerns, all reactions involving the preparation of NTO were performed in a chemical fume hood behind a shatterproof sash and a portable protective shield. TO (12 g, 140 mmol) was added in small aliquots to fuming nitric acid (60 mL) at 0 ºC in an ice bath. After the

TO was added completely over about 10 min, the ice bath was removed, and the reaction mixture was heated to 70 °C and kept for 16 h. An exothermic reaction occurred with the evolution of brown fumes and formation of a precipitate. The reaction mixture was cooled in an ice bath, and the precipitate was filtered off and washed with ice cold water to remove excess nitric acid. The crude product was recrystallized from water to give 7.82 g (yield: 42%) NTO as white solid. ^{1}H NMR (400 MHz, DMSO-d_6) δ 12.80 (br s); ^{13}C NMR (100 MHz, DMSO-d_6) δ 154.3, 147.8; HR-MS (ESI): Calc'd for $C_2HN_4O_3$: 129.0049 $[M\text{-}H]^-$, Found: 129.0049 $[M\text{-}H]^-$.

Section S2. Contribution of NTO-derived, hydroxyl-radical mediated degradation of FFA

Overall, little pCBA was degraded in the presence or absence of NTO. While the presence of NTO did slightly enhance the rate of pCBA degradation (p=0.011, paired t-test of initial concentration-normalized data), the steady-state concentration of hydroxyl radicals was calculated to be only 3×10^{-16} M using the equation:

$$[R]_{ss} = \frac{k_{obs}^{probe}}{k_{OH,1O2}} \quad (1)$$

where $[R]_{ss}$ is the concentration of radical species in M, k_{obs} is the observed reaction rate of the probe compound, and $k_{OH,1O2}$ is the known second-order reaction rate of the probe compound. FFA is known to react with hydroxyl radical at the rate of 1.5×10^{10} M^{-1} s^{-1}, but the concentration of $^{\bullet}OH$ determined from the pCBA experiment would only result in minor FFA degradation (i.e., $k_{obs,FFA}/k_{calc,OH,FFA} = \sim 12$).[1]

Section S3. Light screening and quantum yield calculations

Indirect photolysis rates and solutions with 5 mM 2,4-hexadienoic acid (sorbic acid) were corrected for light screening as follows.[2] UV-Vis spectra of SRNOM and sorbic acid solutions were collected on a spectrophotometer (Hach, Loveland, CO) in a quartz cuvette with a path length of 1 cm. We then calculated the wavelength-dependent screening factor (S_λ) according to the following equation:

$$S_\lambda = \frac{1 - 10^{-A_\lambda}}{2.303 A_\lambda}$$

where A_λ is the solution absorbance. We summed the light intensity from the solar simulator over 290 – 455 nm (L_{total}) and corrected the observed rates for solution screening factor according to the following equation:

$$k_{obs,corr} = \frac{k_{obs}}{\sum_{\lambda=290}^{455} \frac{S_\lambda L_\lambda}{L_{total}}}$$

where $k_{obs,corr}$ is the corrected pseudo-first order rate constant, k_{obs} is the observed pseudo-first order rate constant, and L_λ is the light intensity at the respective wavelength.

Quantum yield was calculated using the absorption spectrum for *para*-nitroanisole (PNA), ε_{PNA}, and the updated quantum yield equation for the PNA-pyridine system from Laszakovits, et. al:

$$\Phi_{PNA} = [pyr] + 0.00029$$

where Φ_{PNA} is the quantum yield of PNA and [pyr] is the concentration of pyridine.[3] Quantum yield of NTO, Φ, was then calculated as:

$$\Phi = \frac{k_{NTO}}{k_{PNA}} \frac{\sum_{\lambda} L_{\lambda} \varepsilon_{\lambda,PNA} \Delta\lambda}{\sum_{\lambda} L_{\lambda} \varepsilon_{\lambda,NTO} \Delta\lambda} \Phi_{PNA}$$

where k_i is the photolysis rate constant for the compound, L_λ is the irradiance of the light source, and $\varepsilon_{\lambda,i}$ is the absorbance of the compound, and Δλ is the wavelength interval (1 nm for these data).[4]

Section S4. Singlet oxygen and hydroxyl radical second-order rate constants

To determine the second-order reaction rate constant of NTO with $^1O_2$, $k_{^1O_2}$, we simultaneously photolyzed 20 µM solutions of either NTO or furfuryl alcohol (FFA) containing 5 µM Rose Bengal in 5 mM borate buffer at pH 8.5. Rose Bengal was used as a sensitizer to produce singlet oxygen and FFA has a known reaction rate with singlet oxygen, $k_{^1O_{2,FFA}}$ = 1.273 $\times 10^8$ M^{-1} s^{-1} at 35 °C.[5] We used a 200 W xenon lamp (Oriel Instruments, Irvine CA, USA) with a 400 nm cut off filter (Figure 1D) to minimize direct photolysis, and no degradation occurred in controls without Rose Bengal.

$$\ln\left(\frac{[NTO]_t}{[NTO]_0}\right) = \frac{k_{1O2,NTO}}{k_{1O2,FFA}} \ln\left(\frac{[FFA]_t}{[FFA]_0}\right)$$

To determine the second-order reaction rate constant of NTO with $^\bullet OH$, k_{OH}, we used a competition kinetics approach. We photolyzed a solution containing 10 mM $NaNO_3$ with 10 µM of both NTO and *para*-chlorobenzoic acid (pCBA) in 5 mM phosphate buffer at pH 7. The nitrate produces $^\bullet OH$, while pCBA reacts with a known k_{OH}. The concentration of NTO was corrected for direct photolysis according to Wenk et al.,[6] and $k_{OH,NTO}$ was calculated using the slope of a linear regression of the equation

$$\ln\left(\frac{[NTO]_t}{[NTO]_0}\right) = \frac{k_{OH,NTO}}{k_{OH,pCBA}} \ln\left(\frac{[pCBA]_t}{[pCBA]_0}\right)$$

where $k_{OH,pCBA}$ is 5×10^9 M^{-1} s^{-1}.[7]

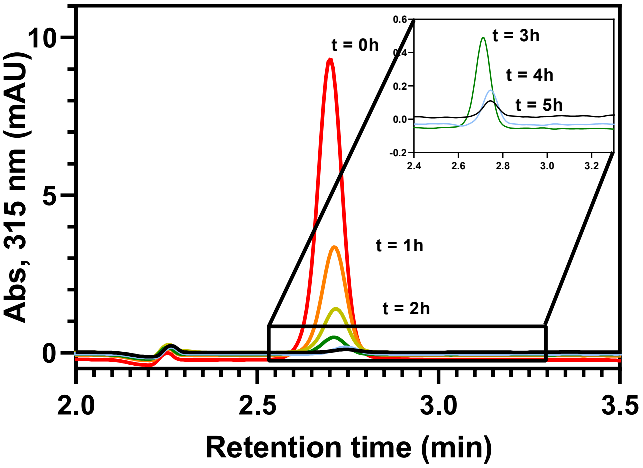

Figure S1. Representative chromatograms depicting NTO detection (using HPLC with UV-DAD monitoring at 315 nm) and degradation through five hours of photolysis (pH 2, 10 μM NTO initial concentration).

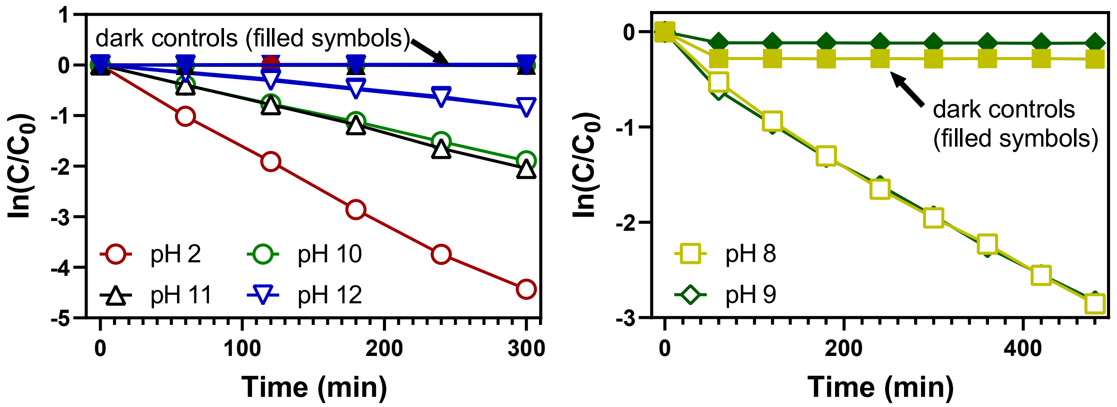

Figure S2. Experimental data comparing illuminated and foil-wrapped dark controls. Data are the natural log of the concentration of NTO at each time point normalized to the initial concentration of NTO.

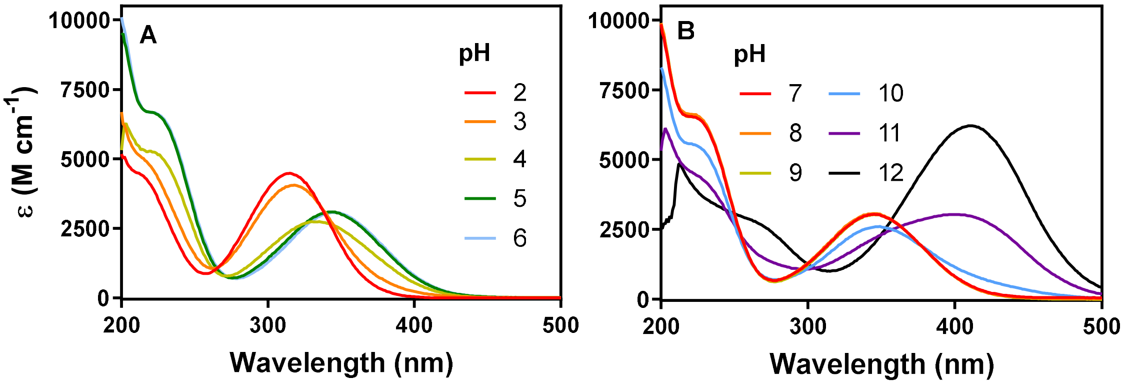

Figure S3. A) molar extinction coefficient of NTO from A) pH 2 to 6 and B) pH 7 to 12 (100 µM NTO), 25 mM buffer solution

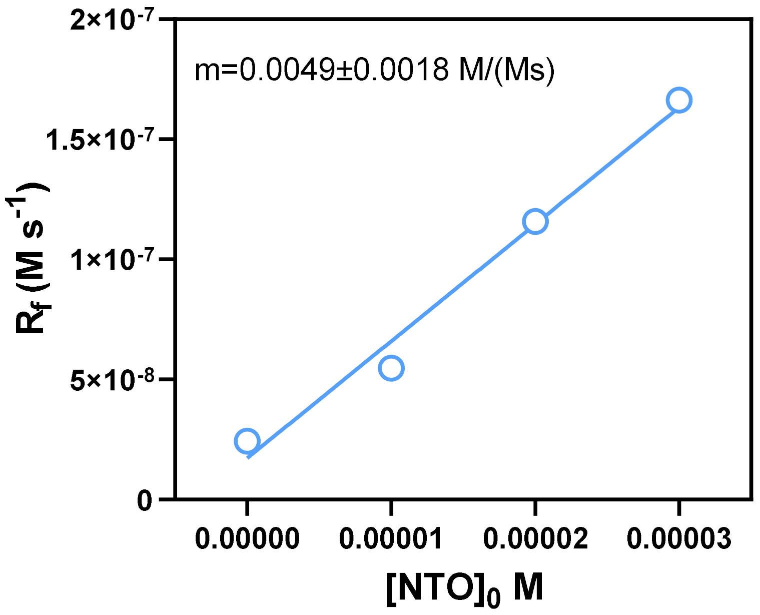

Figure S4. Calculated formation rate of singlet oxygen calculated from FFA degradation rates as a function of initial concentration of NTO.

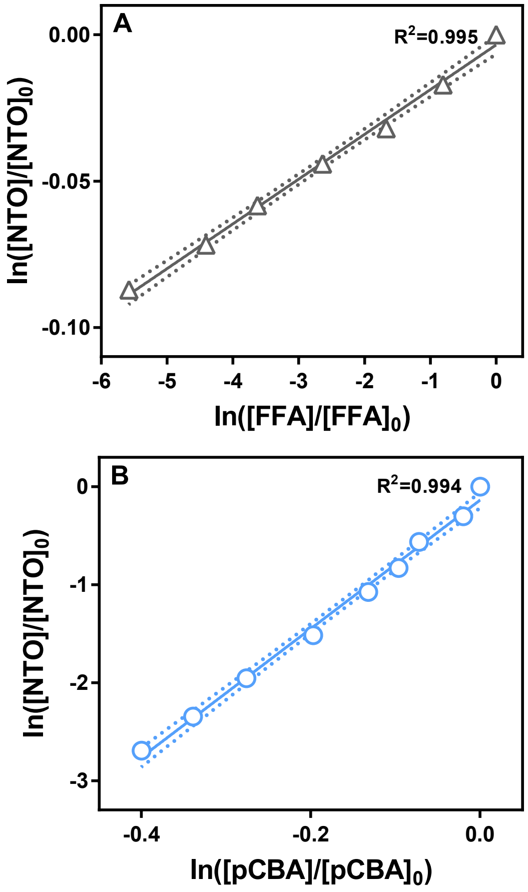

Figure S5. Determination of 2nd-order reaction rate constants of NTO with A) singlet oxygen using the 200 W xenon lamp with a 400 nm cut off filter to minimize direct photolysis, Rose Bengal to produce singlet oxygen, and FFA as a probe and B) hydroxyl radical using competition kinetics with pCBA as a probe and nitrate as a sensitizer.[7] The estimated 2nd-order, bimolecular reaction rate constant of NTO with singlet oxygen was 2.0×10^6 M^{-1} $s^{-1}$and with hydroxyl radicals was 3.3×10^{10} M^{-1} s^{-1}. Solid lines are linear regression and dotted lines are 95% confidence intervals.

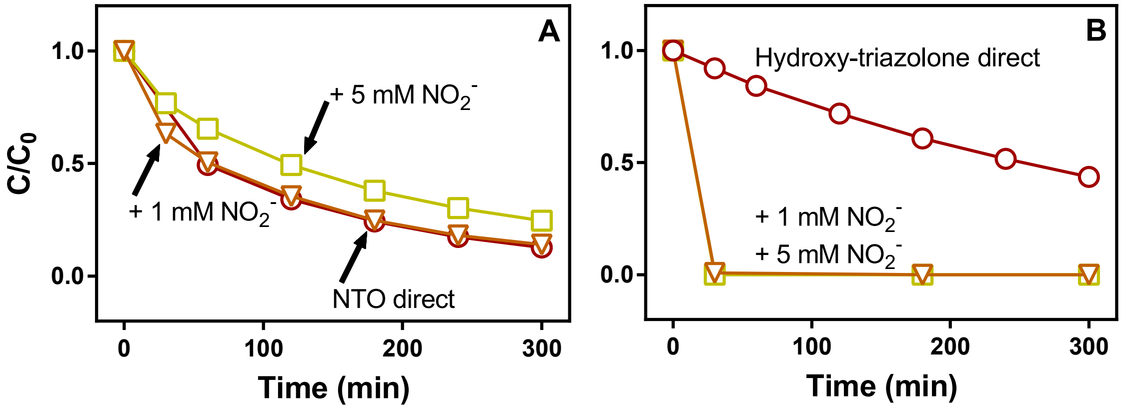

Figure S6. Effect of nitrite on 10 μM A) NTO and B) hydroxyl-triazolone photolysis (25 mM, pH 9 borate buffer, n=1).

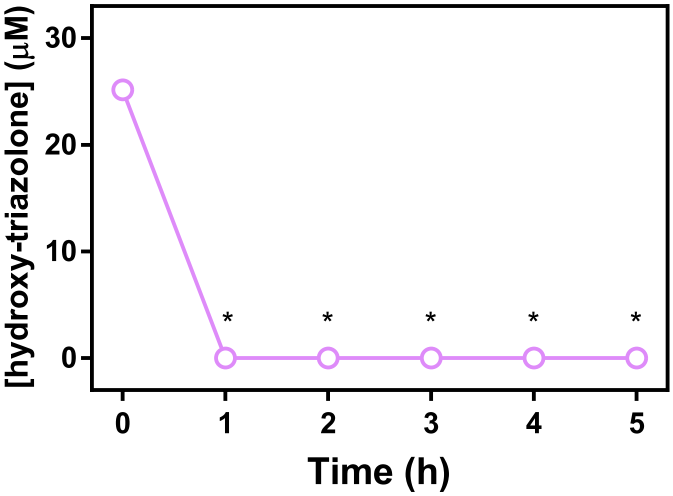

Figure S7. Indirect photolysis of hydroxyl-triazolone (25 μM, 25 mM pH 9 borate buffer, n=1) with 10 μM Rose Bengal to generate singlet oxygen under a 200W Xe-Hg lamp with a 400 nm cut off filter. Asterisks indicate non-detect (LOD < 0.1 μM).

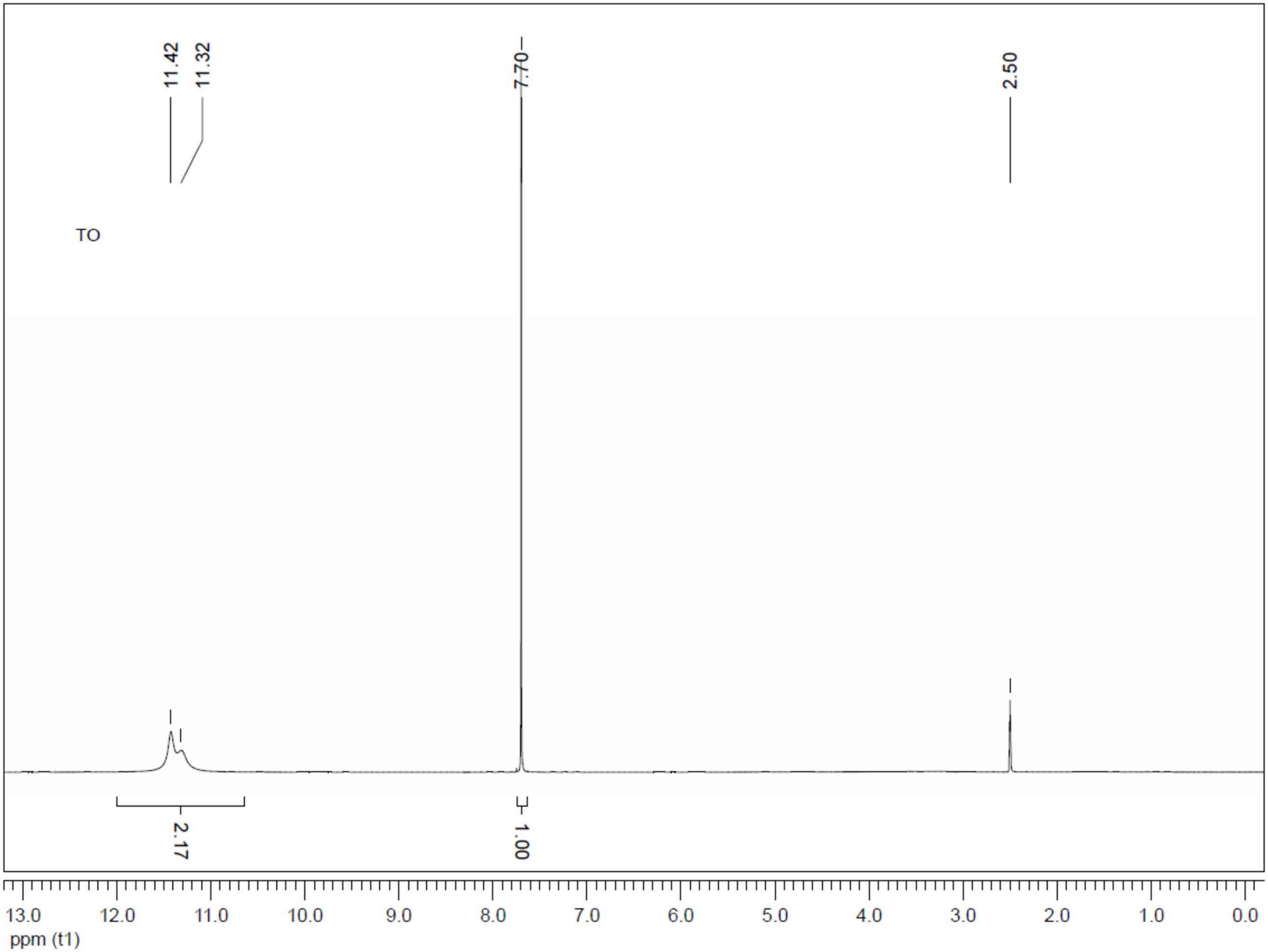

Figure S8. ^{1}H NMR of 1,2,4-triazolone (TO)

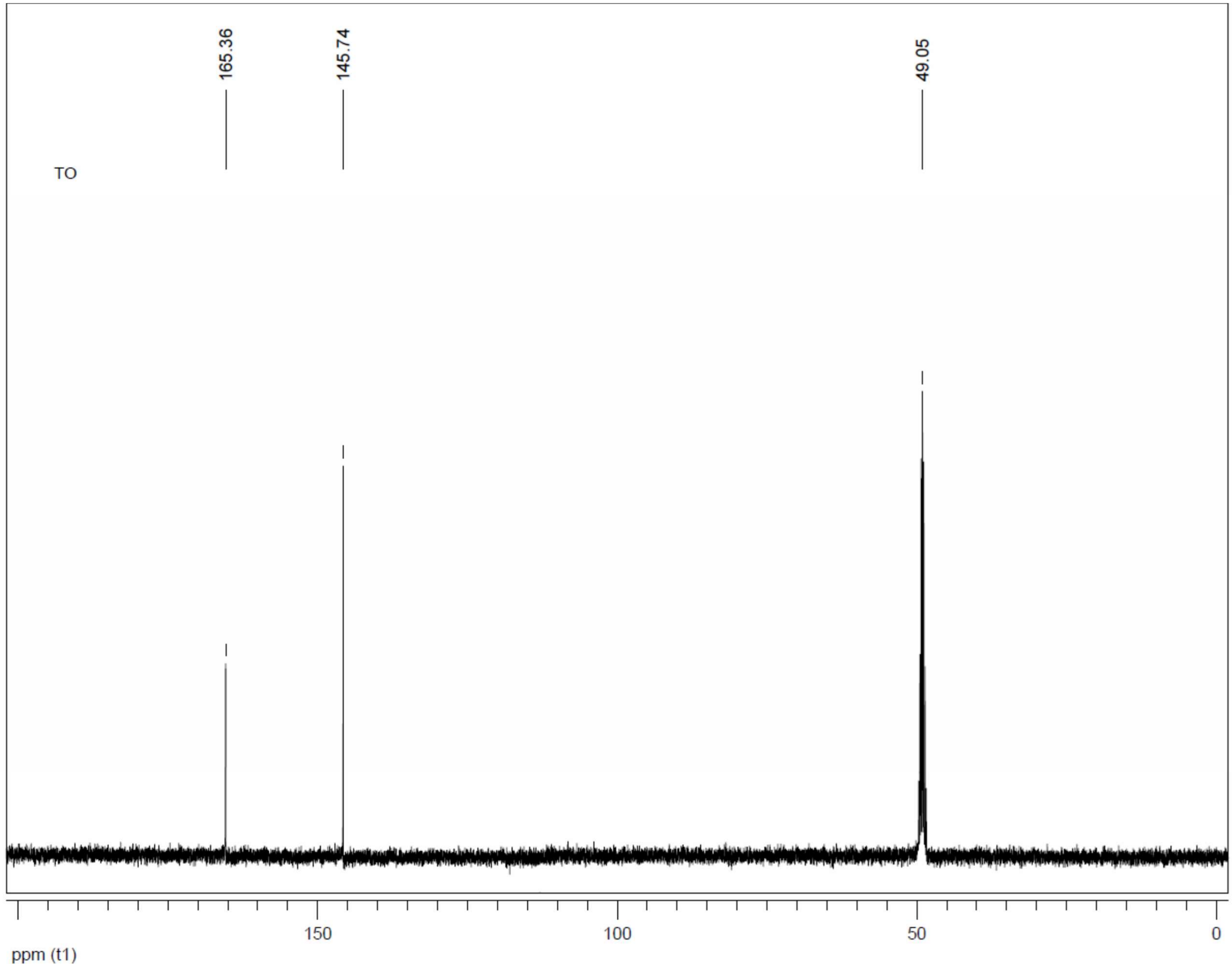

Figure S9. ^{13}C NMR of 1,2,4-triazolone (TO)

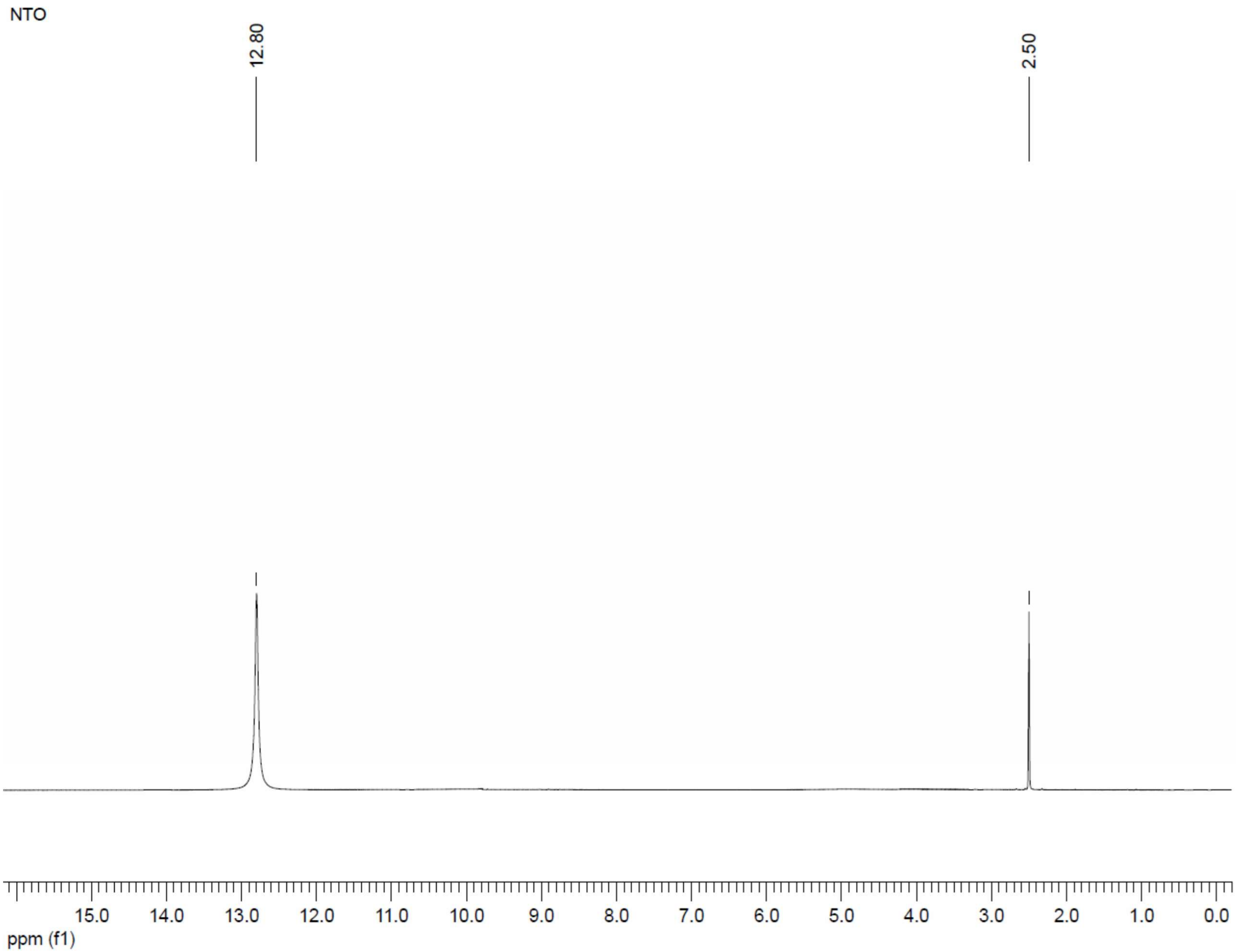

Figure S10. ^{1}H NMR of 3-nitro-1,2,4-triazol-5-one (NTO)

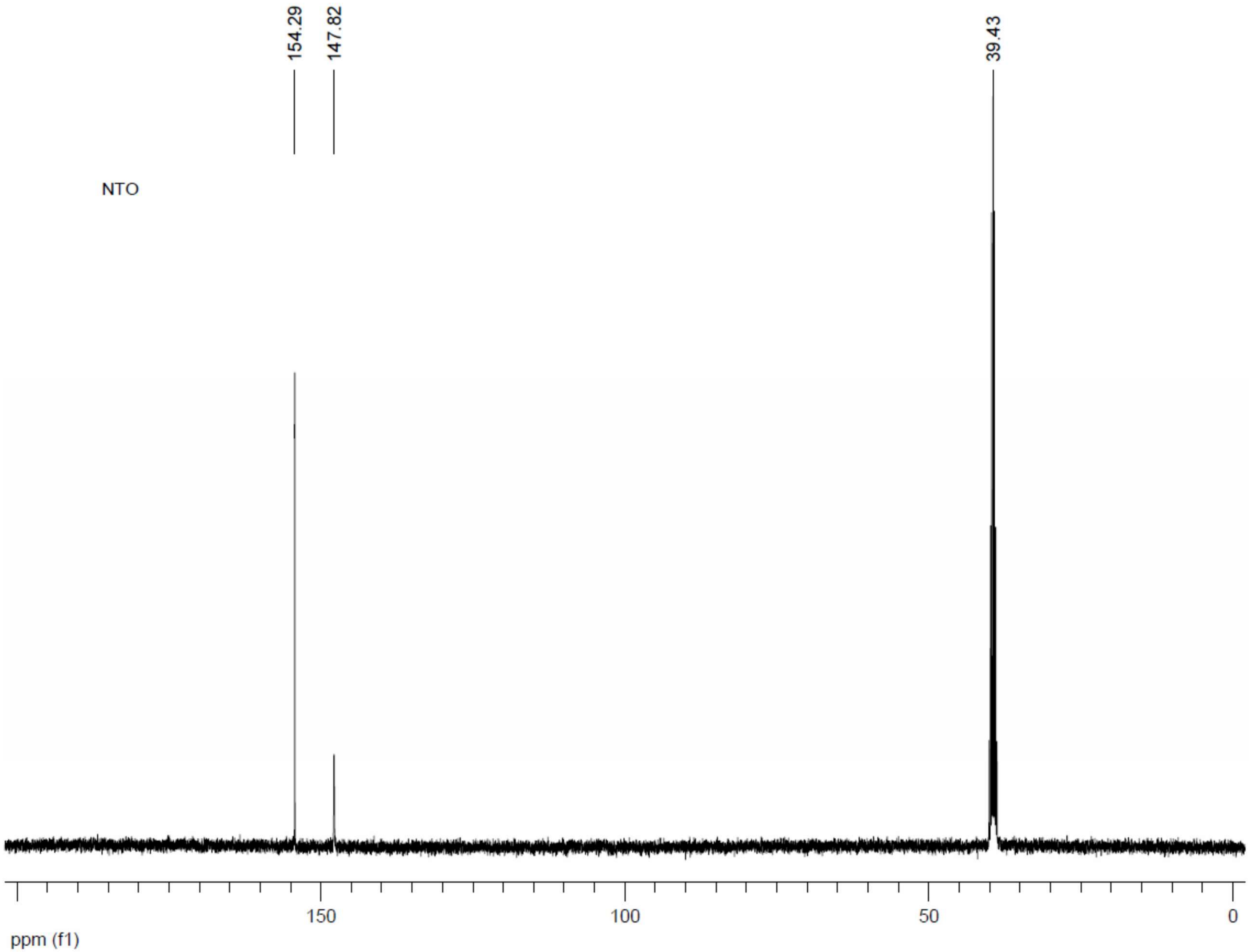

Figure S11. ^{13}C NMR of 3-nitro-1,2,4-triazol-5-one (NTO)

Elemental Composition Report

Single Mass Analysis

Tolerance = 5.0 mDa / DBE: min = -1.5, max = 100.0
Element prediction: Off
Number of isotope peaks used for i-FIT = 3

Monoisotopic Mass, Even Electron Ions
86 formula(e) evaluated with 3 results within limits (all results (up to 1000) for each mass)
Elements Used:
C: 0-120 H: 0-200 N: 0-20 O: 0-20

Lehmler/Li NTO
T03311504 22 (0.441) Cm (21:34) 1: TOF MS ES-
1.14e+004

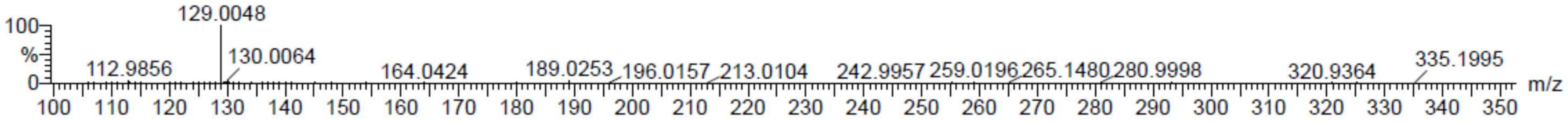

		mDa	PPM	DBE			
Minimum:				-1.5			
Maximum:		5.0	5.0	100.0			

Mass	Calc. Mass	mDa	PPM	DBE	i-FIT	i-FIT (Norm)	Formula
129.0049	129.0049	0.0	0.0	4.5	398.4	0.0	C2 H N4 O3
	129.0035	1.4	10.9	-0.5	401.8	3.3	C H5 O7
	129.0089	-4.0	-31.0	8.5	403.2	4.8	C7 H N2 O

Figure S12. Example high resolution mass spectra and elemental composition report of synthesized 3-nitro-1,2,4-triazol-5-one (NTO). Report was an average of three mass spectra (*m/z* 129.0048, 129.0050, and 129.0050).

References

1 Chu, C. *et al.* Photochemical and Nonphotochemical Transformations of Cysteine with Dissolved Organic Matter. *Environmental Science & Technology* **50**, 6363-6373 (2016). https://doi.org:10.1021/acs.est.6b01291

2 McConville, M. B., Hubert, T. D. & Remucal, C. K. Direct Photolysis Rates and Transformation Pathways of the Lampricides TFM and Niclosamide in Simulated Sunlight. *Environmental Science & Technology* **50**, 9998-10006 (2016). https://doi.org:10.1021/acs.est.6b02607

3 Laszakovits, J. R. *et al.* p-Nitroanisole/Pyridine and p-Nitroacetophenone/Pyridine Actinometers Revisited: Quantum Yield in Comparison to Ferrioxalate. *Environmental Science & Technology Letters* **4**, 11-14 (2017). https://doi.org:10.1021/acs.estlett.6b00422

4 Boreen, A. L., Arnold, W. A. & McNeill, K. Photochemical Fate of Sulfa Drugs in the Aquatic Environment: Sulfa Drugs Containing Five-Membered Heterocyclic Groups. *Environmental Science & Technology* **38**, 3933-3940 (2004). https://doi.org:10.1021/es0353053

5 Appiani, E., Ossola, R., Latch, D. E., Erickson, P. R. & McNeill, K. Aqueous singlet oxygen reaction kinetics of furfuryl alcohol: effect of temperature, pH, and salt content. *Environmental Science: Processes & Impacts* **19**, 507-516 (2017). https://doi.org:10.1039/C6EM00646A

6 Wenk, J., von Gunten, U. & Canonica, S. Effect of Dissolved Organic Matter on the Transformation of Contaminants Induced by Excited Triplet States and the Hydroxyl Radical. *Environmental Science & Technology* **45**, 1334-1340 (2011). https://doi.org:10.1021/es102212t

7 Prasse, C., Wenk, J., Jasper, J. T., Ternes, T. A. & Sedlak, D. L. Co-occurrence of Photochemical and Microbiological Transformation Processes in Open-Water Unit Process Wetlands. *Environmental Science & Technology* **49**, 14136-14145 (2015). https://doi.org:10.1021/acs.est.5b03783
